# Publisher Correction: Ganglioglioma deep transcriptomics reveals primitive neuroectoderm neural precursor‑like population

**DOI:** 10.1186/s40478-024-01788-x

**Published:** 2024-06-27

**Authors:** Joshua A. Regal, María E. Guerra García, Vaibhav Jain, Vidyalakshmi Chandramohan, David M. Ashley, Simon G. Gregory, Eric M. Thompson, Giselle Y. López, Zachary J. Reitman

**Affiliations:** 1 Department of Radiation Oncology, Duke University, Durham, NC 27710 USA; 2 Duke Molecular Physiology Institute, Duke University, Durham, NC 27710 USA; 3 Department of Neurosurgery, Duke University, Durham, NC 27710 USA; 4 Department of Pathology, Duke University, Durham, NC 27710 USA

**Publisher Correction: Acta Neuropathologica Communications (2023) 11:50** 10.1186/s40478-023-01548-3

Following the publication of the original article [1], it was noted that due to a typesetting error Fig. 10 was incorrect. A part of Fig. 6 was incorrectly included also as Fig. 10. The correct figure is given hereafter.

The publisher apologizes for the inconvenience caused.

The incorrect Fig. 10 reads:

**Fig. 10:**
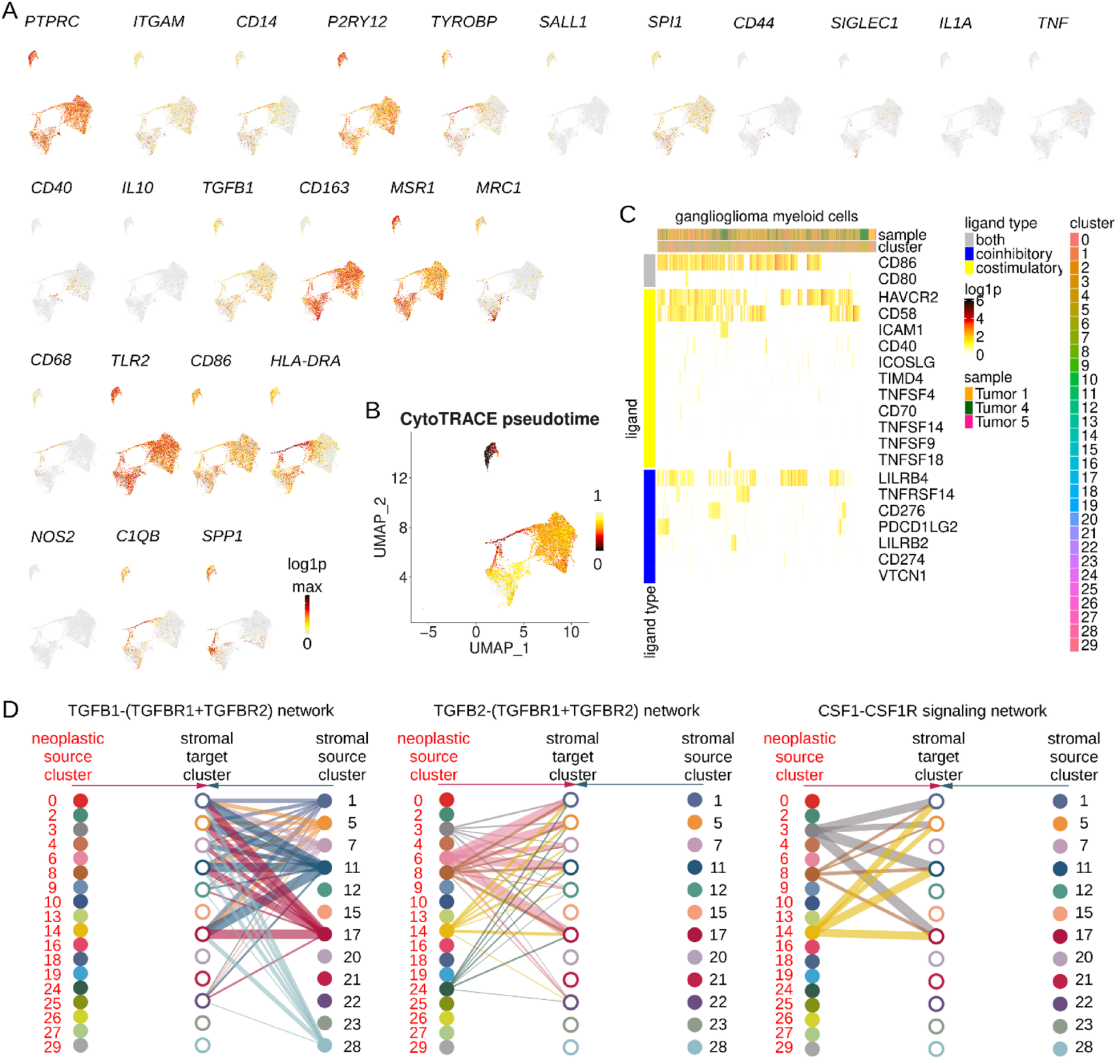
Low grade glioma outcomes analysis. **A, B** Kaplan–Meier EFS (95% CI shaded, dotted line for median) for Bergthold et al. patients overall (**A**) and by resection status (**B**). **C** Kaplan–Meier EFS (95% CI shaded, dotted line for median) for Bergthold et al. patients by UCell score quartile for the gene signature of CD34, SOX2, CD99, and CTSC. **D** Forest plot of COXPH EFS HR for Bergthold et al. patients with continuous variables kept continuous. Includes HR for Z-score for the gene signature of CD34, SOX2, CD99, and CTSC. EFS = event-free survival, NR = none reported, GTR = gross total resection, NTR = near total resection, G/NTR = gross or near total resection, STR = subtotal resection, WT = wild-type. DA = diffuse astrocytoma, GG = ganglioglioma, ODG = oligodendroglioma, DNT = dysplastic neuroepithelial tumor, NOS or LGG = low-grade glioma, not otherwise specified. PA = pilocytic astrocytoma

The correct Fig. 10 should read:

**Fig. 10:**
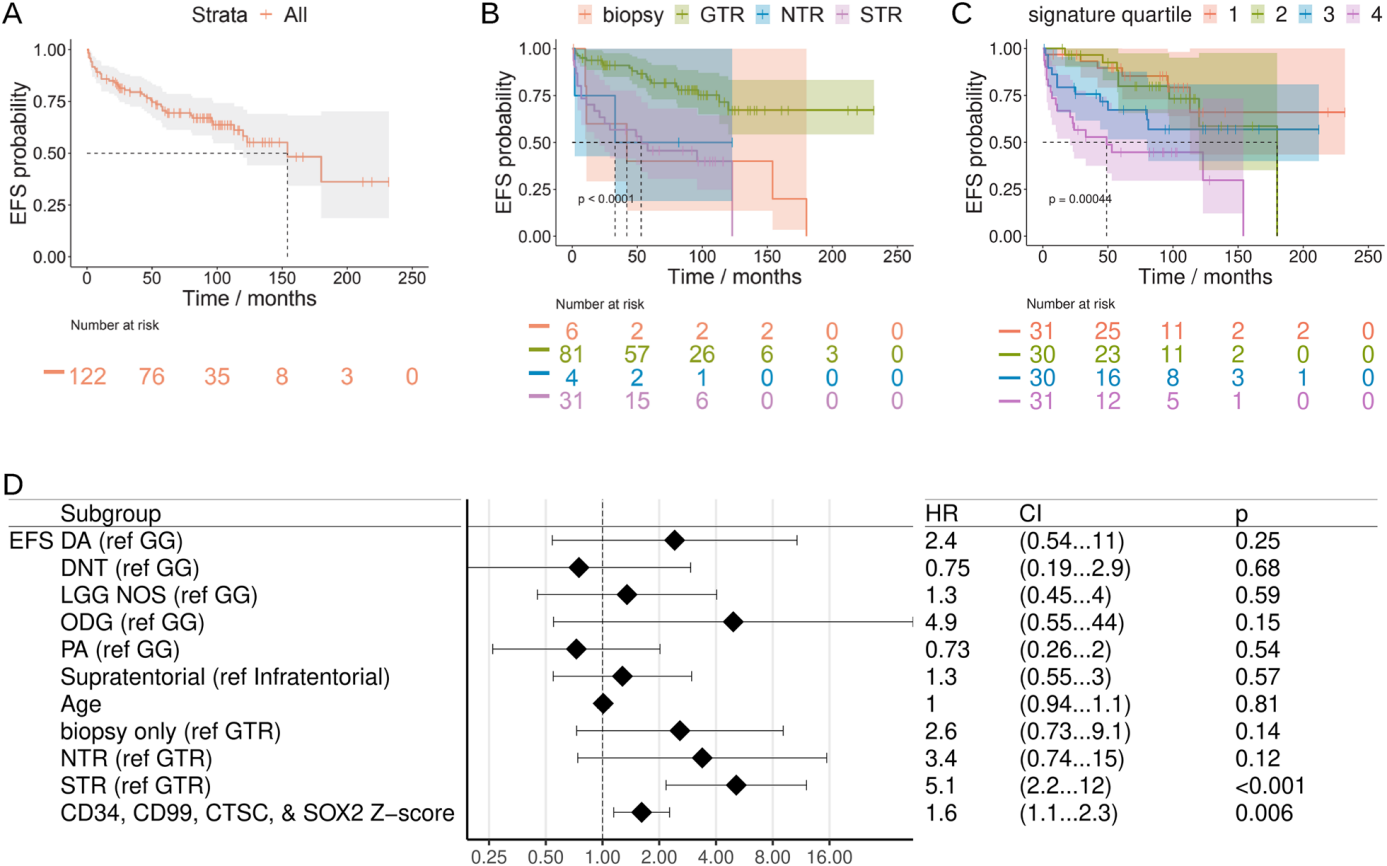
Low grade glioma outcomes analysis. **A, B** Kaplan–Meier EFS (95% CI shaded, dotted line for median) for Bergthold et al. patients overall (**A**) and by resection status (**B**). **C** Kaplan–Meier EFS (95% CI shaded, dotted line for median) for Bergthold et al. patients by UCell score quartile for the gene signature of CD34, SOX2, CD99, and CTSC. **D** Forest plot of COXPH EFS HR for Bergthold et al. patients with continuous variables kept continuous. Includes HR for Z-score for the gene signature of CD34, SOX2, CD99, and CTSC. EFS = event-free survival, NR = none reported, GTR = gross total resection, NTR = near total resection, G/NTR = gross or near total resection, STR = subtotal resection, WT = wild-type. DA = diffuse astrocytoma, GG = ganglioglioma, ODG = oligodendroglioma, DNT = dysplastic neuroepithelial tumor, NOS or LGG = low-grade glioma, not otherwise specified. PA = pilocytic astrocytoma.

The correct figure has been included in this correction, and the original article [1] has been corrected.
